# The genome sequence of the Jersey Tiger moth, *Euplagia quadripunctaria *(Poda, 1761)

**DOI:** 10.12688/wellcomeopenres.23666.1

**Published:** 2025-02-10

**Authors:** David C. Lees, Peter W. H. Holland, Natural History Museum Genome Acquisition Lab, Darwin Tree of Life Barcoding collective, Wellcome Sanger Institute Tree of Life Management, Samples and Laboratory team, Wellcome Sanger Institute Scientific Operations: Sequencing Operations, Wellcome Sanger Institute Tree of Life Core Informatics team, Tree of Life Core Informatics collective, Darwin Tree of Life Consortium

**Affiliations:** 1 Natural History Museum, London, England, UK; 2 University of Oxford, Oxford, England, UK

**Keywords:** Euplagia quadripunctaria, Jersey Tiger, genome sequence, chromosomal, Lepidoptera

## Abstract

We present a genome assembly from an individual male specimen of *Euplagia quadripunctaria* (Jersey Tiger; Arthropoda; Insecta; Lepidoptera; Erebidae). The genome sequence has a total length of 668.40 megabases. Most of the assembly (99.6%) is scaffolded into 31 chromosomal pseudomolecules, including the Z sex chromosome. The mitochondrial genome has also been assembled and is 15.48 kilobases in length.

## Species taxonomy

Eukaryota; Opisthokonta; Metazoa; Eumetazoa; Bilateria; Protostomia; Ecdysozoa; Panarthropoda; Arthropoda; Mandibulata; Pancrustacea; Hexapoda; Insecta; Dicondylia; Pterygota; Neoptera; Endopterygota; Amphiesmenoptera; Lepidoptera; Glossata; Neolepidoptera; Heteroneura; Ditrysia; Obtectomera; Noctuoidea; Erebidae; Arctiinae; Callimorphini; *Euplagia*; *Euplagia quadripunctaria* (Poda, 1761) (NCBI:txid987932)

## Background

*Euplagia quadripunctaria* (Poda, 1761), also known as the Jersey Tiger Moth, is a moth in family Erebidae, subfamily Arctiinae, with a wingspan of 52–65 mm and forewing length of 28–33 mm ( Waring *et al.*, 2017). Its forewings are black with diagonal creamy white stripes, including a distinctive Y-shaped stripe near the termen, while the hindwings are typically orange or occasionally yellow. The species is easily recognised and rests in a triangular posture.

The Jersey Tiger is resident in the southern parts of the United Kingdom, including the Channel Islands. Whilst the range is expanding, it has not yet reached Scotland or Ireland. It is very widespread in Europe from the southern and eastern Mediterranean to northern Europe with relatively few records for Southern Scandinavia, east to the Urals and south to the Caspian Sea ( GBIF Secretariat, 2024). In the UK, the moth was established around the River Exe estuary in 1871 ( Brakefield & Liebert, 1985) and has since undergone significant range expansion, reaching London by 2004 ( Randle *et al.*, 2019) and Leeds by 2008 ( Waring *et al.*, 2017). The species does well now in suburban and urban environments, including gardens, although it preferred coastal areas in UK in the past. Rarely, it arrives as an immigrant.

Adults fly from mid-July to mid-September in the UK ( Brakefield & Liebert, 1985; Randle *et al.*, 2019), while in Rhodes they emerge in May ( Walker, 1966). Unlike Mediterranean populations, UK populations do not aestivate. In the valley of Petaloudes on Rhodes, the moths form dense aggregations, resembling fern mats during aestivation leaves ( Lees & Zilli, 2019). Eggs, often laid in batches exceeding 500, hatch after two weeks. Larvae overwinter among dead leaves, feeding on herbaceous plants such as nettles ( *Urtica dioica*), dead nettles ( *Lamium album*), and bramble ( *Rubus fruticosus* agg.) ( Waring *et al.*, 2017). Adults nectar on wildflowers like hemp agrimony ( *Eupatorium cannabinum*) and are active primarily at night, with females releasing pheromones to attract males when temperatures exceed 15 °C ( Brakefield & Liebert, 1985).

The mitogenome from the genomic assembly (OY982590) (BOLD:) is identical to many haplotypes from Europe and belongs to the Barcode Index Number cluster BOLD:AAD9583. There are two main evolutionary lineages and potential sibling species, the latter being known as *Euplagia splendidior* (Tams, 1922) and occurs in Armenia ( Lees & Zilli, 2019: 160–161), Turkey and Iraq. The latter belongs to the same BIN on BOLD but a sample from Iran (VNLEP00129, identical to KX050236: see Ronka *et al*., 2016, Figure 2) is about 2% distant (BOLD, 1/12/2024). The most closely related species to this sibling pair in the sampling of Ronka *et al*. (2016) in the tribe Callimorphini was the Scarlet Tiger *Callimorpha dominula* L.

Colour polymorphism in *E. quadripunctaria* has been studied extensively ( Liebert & Brakefield, 1990). The genome will be helpful, for example, in further work on the evolution, genetics, ecology, colour polymorphism and defence mechanisms in *Euplagia quadripunctaria*. The species is likely to be a sensitive indicator of climate change.

## Genome sequence report

**Figure 1.**
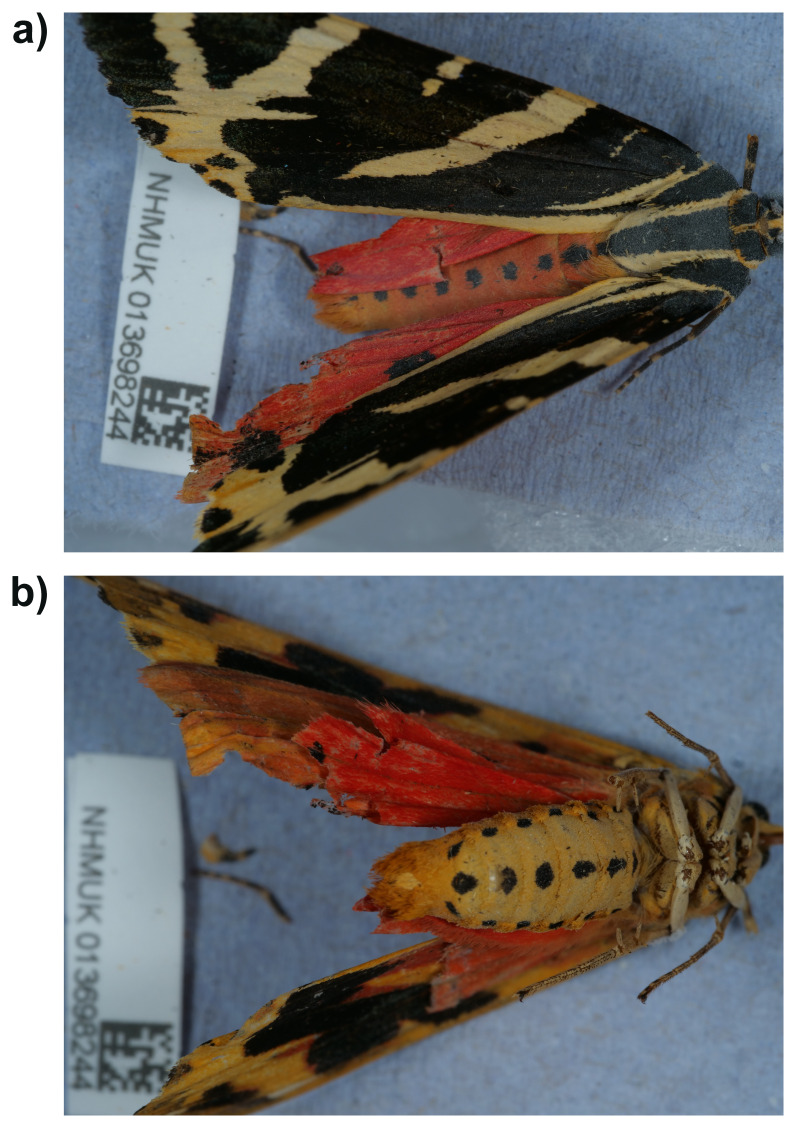
Photographs of the *Euplagia quadripunctaria* (ilEupQuad1) specimen used for genome sequencing. **a**) Dorsal view, **b**) ventral view.

The genome of *Euplagia quadripunctaria* ( Figure 1) was sequenced using Pacific Biosciences single-molecule HiFi long reads, generating a total of 24.61 Gb (gigabases) from 1.94 million reads, providing an estimated 35-fold coverage. Primary assembly contigs were scaffolded with chromosome conformation Hi-C data, which produced 152.80 Gb from 1,011.91 million reads. Specimen and sequencing details are summarised in Table 1.

**Table 1.** Specimen and sequencing data for *Euplagia quadripunctaria*.

Project information			
**Study title**	Euplagia quadripunctaria		
**Umbrella BioProject**	PRJEB64740		
**Species**	*Euplagia quadripunctaria*		
**BioSample**	SAMEA111458330		
**NCBI taxonomy ID**	987932		
Specimen information			
**Technology**	**ToLID**	**BioSample accession**	**Organism part**
**PacBio long read sequencing**	ilEupQuad1	SAMEA111458484	thorax
**Hi-C sequencing**	ilEupQuad1	SAMEA111458518	head
**RNA sequencing**	ilEupQuad3	SAMEA112233256	abdomen
Sequencing information			
**Platform**	**Run accession**	**Read count**	**Base count (Gb)**
**Hi-C Illumina NovaSeq 6000**	ERR11814122	1.01e+09	152.8
**PacBio Sequel IIe**	ERR11809151	1.94e+06	24.61
**RNA Illumina NovaSeq X**	ERR13093640	1.77e+08	26.8

Assembly errors were corrected by manual curation, including 181 missing joins or mis-joins and 24 haplotypic duplications. This reduced the scaffold number by 31.88% and increased the scaffold N50 by 2.43%. The final assembly has a total length of 668.40 Mb in 187 sequence scaffolds, with 1,756 gaps, and a scaffold N50 of 23.2 Mb ( Table 2).

**Table 2.** Genome assembly data for *Euplagia quadripunctaria*, ilEupQuad1.1.

Genome assembly		
Assembly name	ilEupQuad1.1	
Assembly accession	GCA_963859945.1	
*Accession of alternate haplotype*	*GCA_963859955.1*	
Span (Mb)	668.40	
Number of contigs	1,944	
Number of scaffolds	187	
Longest scaffold (Mb)	30.21	
Assembly metrics *		*Benchmark*
Contig N50 length (Mb)	0.7	*≥ 1 Mb*
Scaffold N50 length (Mb)	23.2	*= chromosome N50*
Consensus quality (QV)	59.4	*≥ 40*
*k*-mer completeness	Primary: 88.34%; alternate: 65.85%; combined: 99.38%	*≥ 95%*
BUSCO **	C:96.1%[S:95.4%,D:0.7%], F:0.8%,M:3.1%,n:5,286	*S > 90%, D < 5%*
Percentage of assembly mapped to chromosomes	99.6%	*≥ 90%*
Sex chromosomes	Z	*localised homologous* *pairs*
Organelles	Mitochondrial genome: 15.48 kb	*complete single alleles*

* Assembly metric benchmarks are adapted from Rhie *et al.* (2021) and the Earth BioGenome Project Report on Assembly Standards September 2024. ** BUSCO scores based on the lepidoptera_odb10 BUSCO set using version 5.4.3. C = complete [S = single copy, D = duplicated], F = fragmented, M = missing, n = number of orthologues in comparison. A full set of BUSCO scores is available at https://blobtoolkit.genomehubs.org/view/Euplagia_quadripunctaria/dataset/GCA_963859945.1/busco.

**Figure 2.**
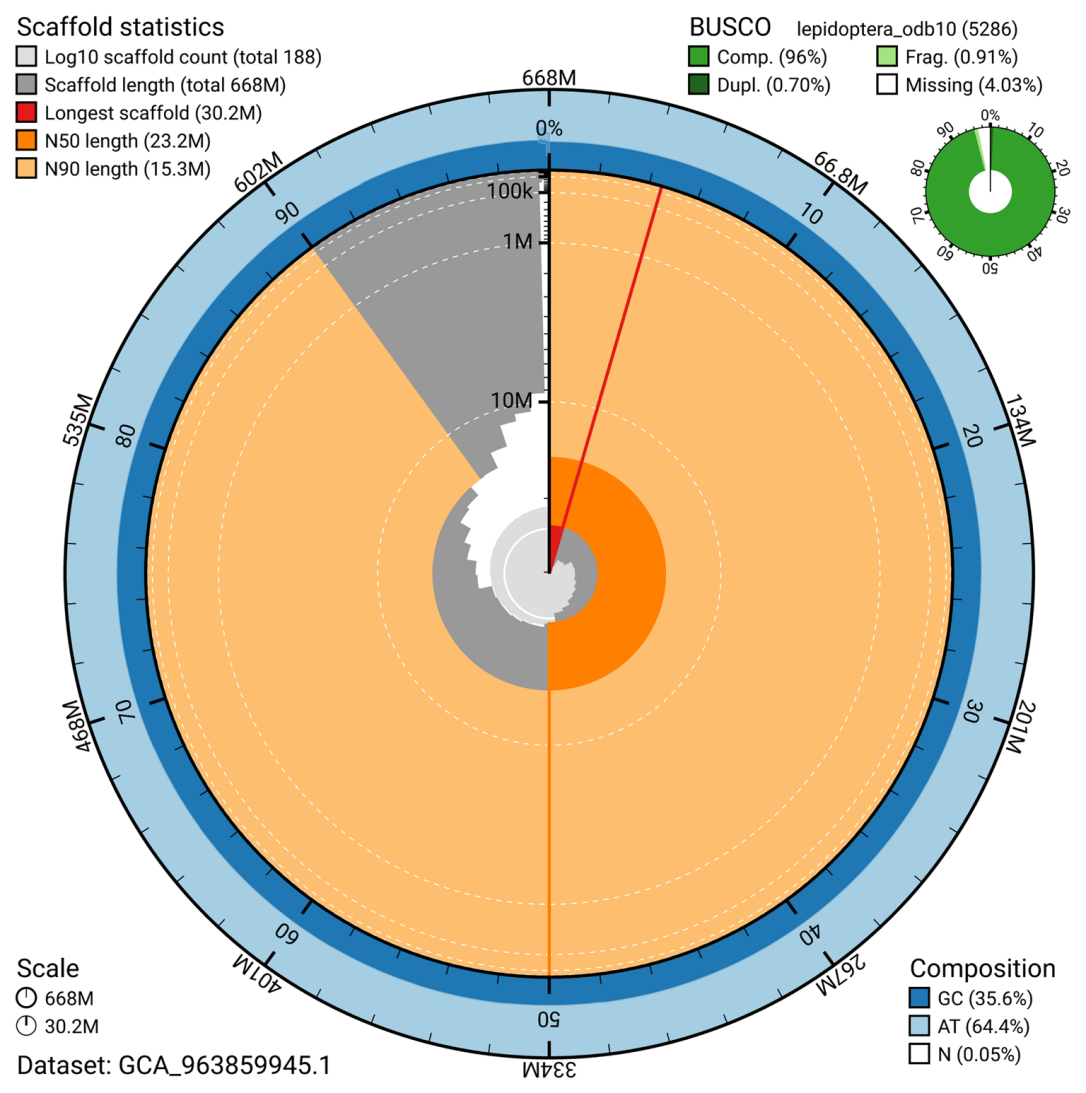
Genome assembly of *Euplagia quadripunctaria*, ilEupQuad1.1: metrics. The BlobToolKit snail plot provides an overview of assembly metrics and BUSCO gene completeness. The circumference represents the length of the whole genome sequence, and the main plot is divided into 1,000 bins around the circumference. The outermost blue tracks display the distribution of GC, AT, and N percentages across the bins. Scaffolds are arranged clockwise from longest to shortest and are depicted in dark grey. The longest scaffold is indicated by the red arc, and the deeper orange and pale orange arcs represent the N50 and N90 lengths. A light grey spiral at the centre shows the cumulative scaffold count on a logarithmic scale. A summary of complete, fragmented, duplicated, and missing BUSCO genes in the lepidoptera_odb10 set is presented at the top right. An interactive version of this figure is available at https://blobtoolkit.genomehubs.org/view/GCA_963859945.1/dataset/GCA_963859945.1/snail.

**Figure 3.**
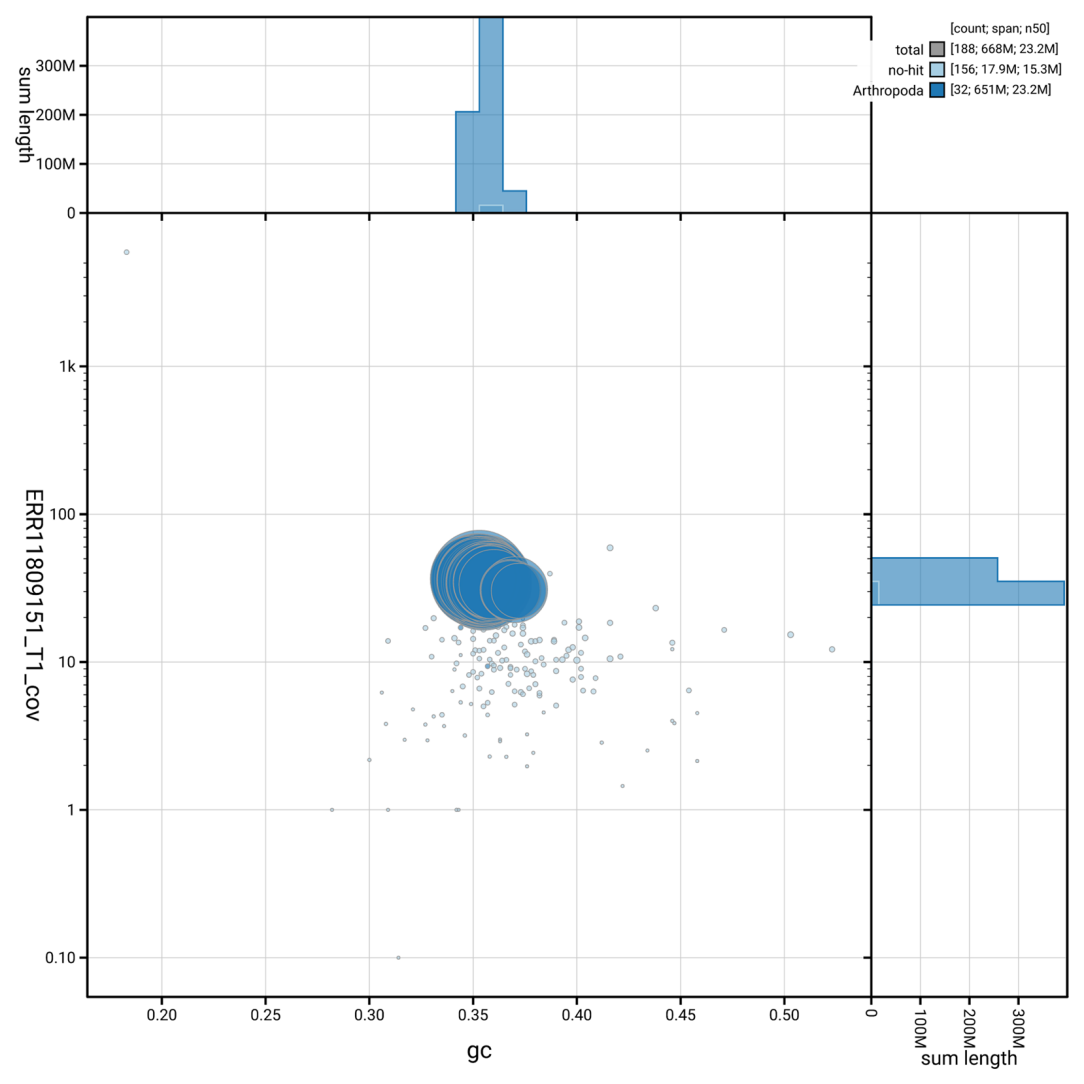
Genome assembly of *Euplagia quadripunctaria*, ilEupQuad1.1: BlobToolKit GC-coverage plot showing sequence coverage (vertical axis) and GC content (horizontal axis). The circles represent scaffolds, with the size proportional to scaffold length and the colour representing phylum membership. The histograms along the axes display the total length of sequences distributed across different levels of coverage and GC content. An interactive version of this figure is available at https://blobtoolkit.genomehubs.org/view/GCA_963859945.1/dataset/GCA_963859945.1/blob.

**Figure 4.**
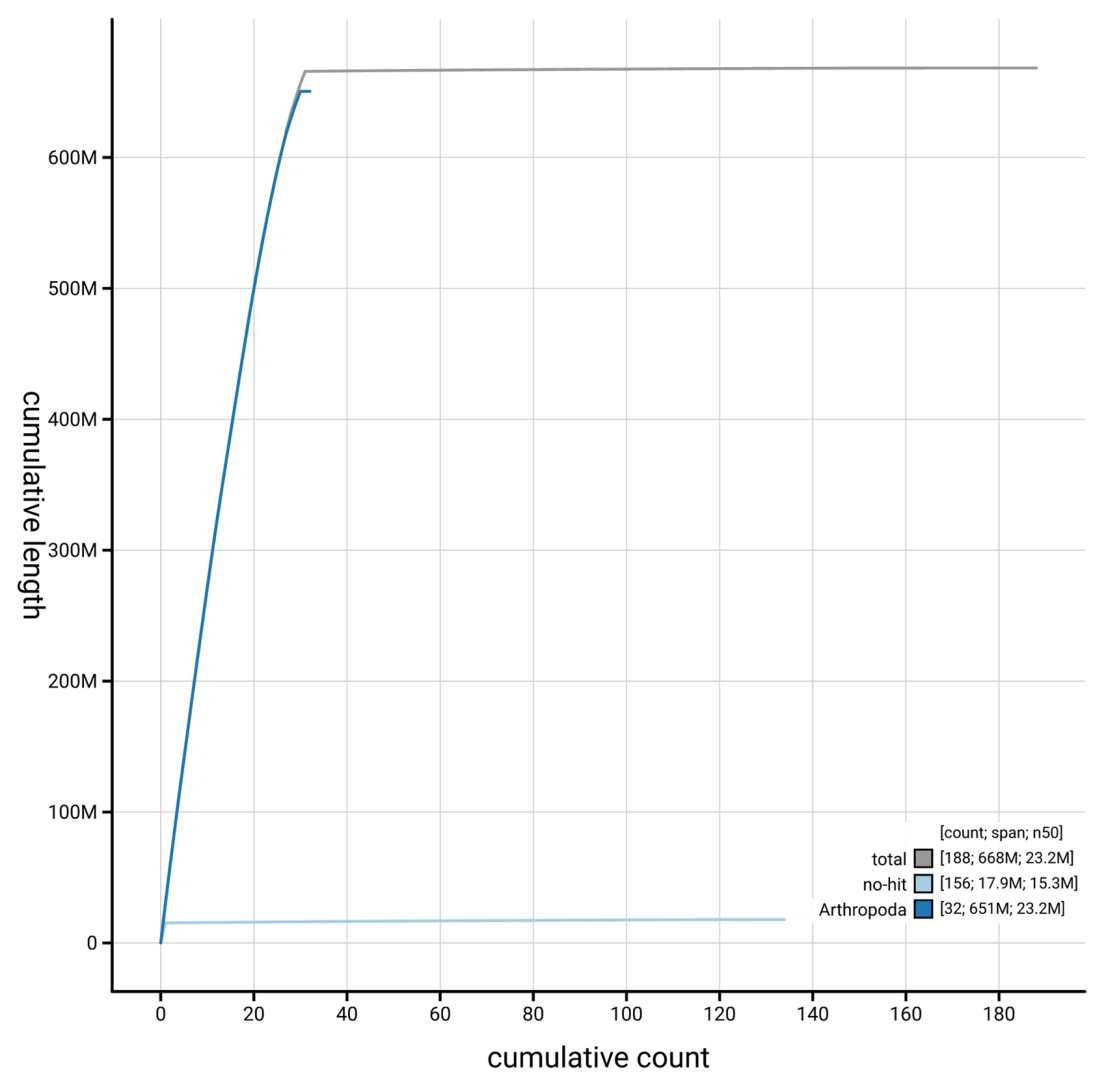
Genome assembly of *Euplagia quadripunctaria* ilEupQuad1.1: BlobToolKit cumulative sequence plot. The grey line shows cumulative length for all scaffolds. Coloured lines show cumulative lengths of scaffolds assigned to each phylum using the buscogenes taxrule. An interactive version of this figure is available at https://blobtoolkit.genomehubs.org/view/GCA_963859945.1/dataset/GCA_963859945.1/cumulative.

The snail plot in Figure 2 provides a summary of the assembly statistics, indicating the distribution of scaffold lengths and other assembly metrics. Figure 3 shows the distribution of scaffolds by GC proportion and coverage. Figure 4 presents a cumulative assembly plot, with separate curves representing different scaffold subsets assigned to various phyla, illustrating the completeness of the assembly.

**Figure 5.**
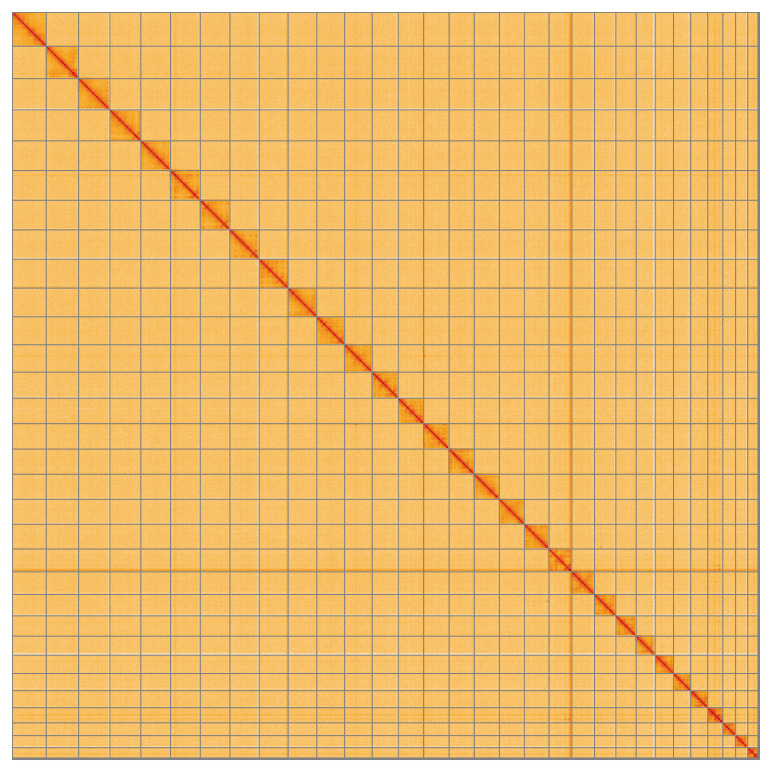
Genome assembly of *Euplagia quadripunctaria* ilEupQuad1.1: Hi-C contact map of the ilEupQuad1.1 assembly, visualised using HiGlass. Chromosomes are shown in order of size from left to right and top to bottom. An interactive version of this figure may be viewed at https://genome-note-higlass.tol.sanger.ac.uk/l/?d=AXfBpQiKRi6AZjQ07j78FQ.

Most of the assembly sequence (99.6%) was assigned to 31 chromosomal-level scaffolds, representing 30 autosomes and the Z sex chromosome. These chromosome-level scaffolds, confirmed by the Hi-C data, are named in order of size ( Figure 5; Table 3). During manual curation, the Z chromosome was assigned by homology to the genome assembly of *Lithosia quadra* (GCA_963576445.1).

**Table 3.** Chromosomal pseudomolecules in the genome assembly of *Euplagia quadripunctaria*, ilEupQuad1.

INSDC accession	Name	Length (Mb)	GC%
OY982560.1	1	29.0	35.5
OY982561.1	2	27.84	35.5
OY982562.1	3	27.65	35.0
OY982563.1	4	26.59	35.5
OY982564.1	5	26.49	35.5
OY982565.1	6	26.48	35.0
OY982566.1	7	26.16	35.5
OY982567.1	8	25.82	35.5
OY982568.1	9	25.52	35.5
OY982569.1	10	24.96	35.5
OY982570.1	11	24.57	35.5
OY982571.1	12	23.2	35.0
OY982572.1	13	22.74	35.5
OY982573.1	14	22.72	35.5
OY982574.1	15	22.48	35.5
OY982575.1	16	22.33	35.5
OY982576.1	17	22.29	35.5
OY982577.1	18	22.1	35.5
OY982578.1	19	20.42	36.0
OY982579.1	20	20.22	36.0
OY982580.1	21	19.04	35.5
OY982581.1	22	18.22	36.0
OY982582.1	23	16.83	36.0
OY982583.1	24	16.49	36.0
OY982584.1	25	15.28	36.0
OY982585.1	26	15.2	36.0
OY982586.1	27	13.5	37.0
OY982587.1	28	11.4	37.0
OY982588.1	29	10.76	37.0
OY982589.1	30	9.25	37.0
OY982559.1	Z	30.21	35.5
OY982590.1	MT	0.02	18.5

While not fully phased, the assembly deposited is of one haplotype. Contigs corresponding to the second haplotype have also been deposited. The mitochondrial genome was also assembled and can be found as a contig within the multifasta file of the genome submission, and as a separate fasta file with accession OY982590.1.

The final assembly has a Quality Value (QV) of 59.4. The *k*-mer completeness was estimated as 88.34% for the primary assembly, 65.85% for the alternate haplotype, and 99.38% for the combined assemblies. BUSCO (v5.4.3) analysis using the lepidoptera_odb10 reference set ( *n* = 5,286) indicated a completeness score of 96.1% (single = 95.4%, duplicated = 0.7%).

## Methods

### Sample acquisition and DNA barcoding

An adult male *Euplagia quadripunctaria* (specimen ID NHMUK013698244, ToLID ilEupQuad1) was collected from Lucas Road, High Wycombe, England, United Kingdom (latitude 51.63, longitude –0.74) on 2021-08-15. The specimen was collected and identified by David Lees (Natural History Museum) and preserved by dry freezing at –80 °C.

The specimen used for RNA sequencing (specimen ID Ox002569, ToLID ilEupQuad3) was an adult specimen collected from Wallingford, Oxfordshire, United Kingdom (latitude 51.6, longitude –1.14) on 2022-07-29, using a light trap. The specimen was collected and identified by Peter Holland (University of Oxford) and preserved on dry ice.

The initial identification was verified by an additional DNA barcoding process according to the framework developed by Twyford *et al*. (2024). A small sample was dissected from the specimens and stored in ethanol, while the remaining parts were shipped on dry ice to the Wellcome Sanger Institute (WSI). The tissue was lysed, the COI marker region was amplified by PCR, and amplicons were sequenced and compared to the BOLD database, confirming the species identification ( Crowley *et al.*, 2023). Following whole genome sequence generation, the relevant DNA barcode region was also used alongside the initial barcoding data for sample tracking at the WSI ( Twyford *et al.*, 2024). The standard operating procedures for Darwin Tree of Life barcoding have been deposited on protocols.io ( Beasley *et al.*, 2023).

Metadata collection for samples adhered to the Darwin Tree of Life project standards described by Lawniczak *et al*. (2022).

### Nucleic acid extraction

The workflow for high molecular weight (HMW) DNA extraction at the Wellcome Sanger Institute (WSI) Tree of Life Core Laboratory includes a sequence of procedures: sample preparation and homogenisation, DNA extraction, fragmentation and purification. Detailed protocols are available on protocols.io ( Denton *et al.*, 2023b). The ilEupQuad1 sample was prepared for DNA extraction by weighing and dissecting it on dry ice ( Jay *et al.*, 2023). Tissue from the thorax was homogenised using a PowerMasher II tissue disruptor ( Denton *et al.*, 2023a). HMW DNA was extracted using the Automated MagAttract v1 protocol ( Sheerin *et al.*, 2023). DNA was sheared into an average fragment size of 12–20 kb in a Megaruptor 3 system ( Todorovic *et al.*, 2023). Sheared DNA was purified by solid-phase reversible immobilisation, using AMPure PB beads to eliminate shorter fragments and concentrate the DNA ( Strickland *et al.*, 2023). The concentration of the sheared and purified DNA was assessed using a Nanodrop spectrophotometer and a Qubit Fluorometer using the Qubit dsDNA High Sensitivity Assay kit. The fragment size distribution was evaluated by running the sample on the FemtoPulse system.

RNA was extracted from abdomen tissue of ilEupQuad3 in the Tree of Life Laboratory at the WSI using the RNA Extraction: Automated MagMax™ *mir*Vana protocol ( do Amaral *et al.*, 2023). The RNA concentration was assessed using a Nanodrop spectrophotometer and a Qubit Fluorometer using the Qubit RNA Broad-Range Assay kit. Analysis of the integrity of the RNA was done using the Agilent RNA 6000 Pico Kit and Eukaryotic Total RNA assay.

### Hi-C sample preparation

Tissue from the head of the ilEupQuad1 sample was processed at the WSI Scientific Operations core, using the Arima-HiC v2 kit. Tissue (stored at –80 °C) was fixed, and the DNA crosslinked using a TC buffer with 22% formaldehyde. After crosslinking, the tissue was homogenised using the Diagnocine Power Masher-II and BioMasher-II tubes and pestles. Following the kit manufacturer's instructions, crosslinked DNA was digested using a restriction enzyme master mix. The 5’-overhangs were then filled in and labelled with biotinylated nucleotides and proximally ligated. An overnight incubation was carried out for enzymes to digest remaining proteins and for crosslinks to reverse. A clean up was performed with SPRIselect beads prior to library preparation.

### Library preparation and sequencing

***PacBio HiFi***

At a minimum, samples were required to have an average fragment size exceeding 8 kb and a total mass over 400 ng to proceed to the low input SMRTbell Prep Kit 3.0 protocol (Pacific Biosciences, California, USA), depending on genome size and sequencing depth required. Libraries were prepared using the SMRTbell Prep Kit 3.0 (Pacific Biosciences, California, USA) as per the manufacturer's instructions. The kit includes the reagents required for end repair/A-tailing, adapter ligation, post-ligation SMRTbell bead cleanup, and nuclease treatment. Following the manufacturer’s instructions, size selection and clean up was carried out using diluted AMPure PB beads (Pacific Biosciences, California, USA). DNA concentration was quantified using the Qubit Fluorometer v4.0 (Thermo Fisher Scientific) with Qubit 1X dsDNA HS assay kit and the final library fragment size analysis was carried out using the Agilent Femto Pulse Automated Pulsed Field CE Instrument (Agilent Technologies) and gDNA 55kb BAC analysis kit.

Samples were sequenced using the Sequel IIe system (Pacific Biosciences, California, USA). The concentration of the library loaded onto the Sequel IIe was between 40–135 pM. The SMRT link software, a PacBio web-based end-to-end workflow manager, was used to set-up and monitor the run, as well as perform primary and secondary analysis of the data upon completion.

***Hi-C***

For Hi-C library preparation, DNA was fragmented using the Covaris E220 sonicator (Covaris) and size selected using SPRISelect beads to 400 to 600 bp. The DNA was then enriched using the Arima-HiC v2 kit Enrichment beads. The NEBNext Ultra II DNA Library Prep Kit (New England Biolabs) was used for end repair, a-tailing, and adapter ligation. This uses a custom protocol which resembles the standard NEBNext Ultra II DNA Library Prep protocol but where library preparation occurs while DNA is bound to the Enrichment beads. For library amplification, 10–16 PCR cycles were required, determined by the sample biotinylation percentage. The Hi-C sequencing was performed using paired-end sequencing with a read length of 150 bp on an Illumina NovaSeq 6000.

***RNA***

Poly(A) RNA-Seq libraries were constructed using the NEB Ultra II RNA Library Prep kit, following the manufacturer’s instructions. RNA sequencing was performed on the Illumina NovaSeq X instrument.

### Genome assembly, curation and evaluation

***Assembly***

The HiFi reads were first assembled using Hifiasm ( Cheng *et al.*, 2021) with the --primary option. Haplotypic duplications were identified and removed using purge_dups ( Guan *et al.*, 2020). The Hi-C reads were mapped to the primary contigs using bwa-mem2 ( Vasimuddin *et al.*, 2019). The contigs were further scaffolded using the provided Hi-C data ( Rao *et al.*, 2014) in YaHS ( Zhou *et al.*, 2023) using the --break option for handling potential misassemblies. The scaffolded assemblies were evaluated using Gfastats ( Formenti *et al.*, 2022), BUSCO ( Manni *et al.*, 2021) and MERQURY.FK ( Rhie *et al.*, 2020).

The mitochondrial genome was assembled using MitoHiFi ( Uliano-Silva *et al.*, 2023), which runs MitoFinder ( Allio *et al.*, 2020) and uses these annotations to select the final mitochondrial contig and to ensure the general quality of the sequence.

***Assembly curation***

The assembly was decontaminated using the Assembly Screen for Cobionts and Contaminants (ASCC) pipeline (article in preparation). Flat files and maps used in curation were generated in TreeVal ( Pointon *et al.*, 2023). Manual curation was primarily conducted using PretextView ( Harry, 2022), with additional insights provided by JBrowse2 ( Diesh *et al.*, 2023) and HiGlass ( Kerpedjiev *et al.*, 2018). Scaffolds were visually inspected and corrected as described by Howe *et al*. (2021). Any identified contamination, missed joins, and mis-joins were corrected, and duplicate sequences were tagged and removed. The sex chromosome was assigned by synteny. The curation process is documented at https://gitlab.com/wtsi-grit/rapid-curation (article in preparation).

***Assembly quality assessment***

The Merqury.FK tool ( Rhie *et al.*, 2020) was used to evaluate *k*-mer completeness and assembly quality for the primary and alternate haplotypes using the *k*-mer databases ( *k* = 31) that were pre-computed prior to genome assembly. The analysis outputs included assembly QV scores and completeness statistics.

A Hi-C contact map was produced for the final, public version of the assembly. The Hi-C reads were aligned using bwa-mem2 ( Vasimuddin *et al.*, 2019) and the alignment files were combined using SAMtools ( Danecek *et al.*, 2021). The Hi-C alignments were converted into a contact map using BEDTools ( Quinlan & Hall, 2010) and the Cooler tool suite ( Abdennur & Mirny, 2020). The contact map is visualised in HiGlass ( Kerpedjiev *et al.*, 2018).

The blobtoolkit pipeline is a Nextflow port of the previous Snakemake Blobtoolkit pipeline ( Challis *et al.*, 2020). It aligns the PacBio reads in SAMtools and minimap2 ( Li, 2018) and generates coverage tracks for regions of fixed size. In parallel, it queries the GoaT database ( Challis *et al.*, 2023) to identify all matching BUSCO lineages to run BUSCO ( Manni *et al.*, 2021). For the three domain-level BUSCO lineages, the pipeline aligns the BUSCO genes to the UniProt Reference Proteomes database ( Bateman *et al.*, 2023) with DIAMOND blastp ( Buchfink *et al.*, 2021). The genome is also divided into chunks according to the density of the BUSCO genes from the closest taxonomic lineage, and each chunk is aligned to the UniProt Reference Proteomes database using DIAMOND blastx. Genome sequences without a hit are chunked using seqtk and aligned to the NT database with blastn ( Altschul *et al.*, 1990). The blobtools suite combines all these outputs into a blobdir for visualisation.

The genome assembly and evaluation pipelines were developed using nf-core tooling ( Ewels *et al.*, 2020) and MultiQC ( Ewels *et al.*, 2016), relying on the Conda package manager, the Bioconda initiative ( Grüning *et al.*, 2018), the Biocontainers infrastructure ( da Veiga Leprevost *et al.*, 2017), as well as the Docker ( Merkel, 2014) and Singularity ( Kurtzer *et al.*, 2017) containerisation solutions.

Table 4 contains a list of relevant software tool versions and sources.

**Table 4.** Software tools: versions and sources.

Software tool	Version	Source
BEDTools	2.30.0	https://github.com/arq5x/bedtools2
BLAST	2.14.0	ftp://ftp.ncbi.nlm.nih.gov/blast/executables/blast+/
BlobToolKit	4.3.7	https://github.com/blobtoolkit/blobtoolkit
BUSCO	5.4.3 and 5.5.0	https://gitlab.com/ezlab/busco
bwa-mem2	2.2.1	https://github.com/bwa-mem2/bwa-mem2
Cooler	0.8.11	https://github.com/open2c/cooler
DIAMOND	2.1.8	https://github.com/bbuchfink/diamond
fasta_ windows	0.2.4	https://github.com/tolkit/fasta_windows
FastK	427104ea91c78c3b8b8b49f1a7d6bbeaa869ba1c	https://github.com/thegenemyers/FASTK
Gfastats	1.3.6	https://github.com/vgl-hub/gfastats
GoaT CLI	0.2.5	https://github.com/genomehubs/goat-cli
Hifiasm	0.19.8-r587	https://github.com/chhylp123/hifiasm
HiGlass	44086069ee7d4d3f6f3f0012569789ec138f42b84aa 44357826c0 b6753eb28de	https://github.com/higlass/higlass
Merqury.FK	d00d98157618f4e8d1a9190026b19b471055b22e	https://github.com/thegenemyers/MERQURY.FK
MitoHiFi	3	https://github.com/marcelauliano/MitoHiFi
MultiQC	1.14, 1.17, and 1.18	https://github.com/MultiQC/MultiQC
NCBI Datasets	15.12.0	https://github.com/ncbi/datasets
Nextflow	23.04.0-5857	https://github.com/nextflow-io/nextflow
PretextView	0.2.5	https://github.com/sanger-tol/PretextView
purge_dups	1.2.5	https://github.com/dfguan/purge_dups
samtools	1.16.1, 1.17, and 1.18	https://github.com/samtools/samtools
sanger-tol/ ascc	-	https://github.com/sanger-tol/ascc
sanger-tol/ blobtoolkit	0.6.0	https://github.com/sanger-tol/blobtoolkit
Seqtk	1.3	https://github.com/lh3/seqtk
Singularity	3.9.0	https://github.com/sylabs/singularity
TreeVal	1.0.0	https://github.com/sanger-tol/treeval
YaHS	1.2a.2	https://github.com/c-zhou/yahs

### Wellcome Sanger Institute – Legal and Governance

The materials that have contributed to this genome note have been supplied by a Darwin Tree of Life Partner. The submission of materials by a Darwin Tree of Life Partner is subject to the **‘Darwin Tree of Life Project Sampling Code of Practice’**, which can be found in full on the Darwin Tree of Life website here. By agreeing with and signing up to the Sampling Code of Practice, the Darwin Tree of Life Partner agrees they will meet the legal and ethical requirements and standards set out within this document in respect of all samples acquired for, and supplied to, the Darwin Tree of Life Project. 

Further, the Wellcome Sanger Institute employs a process whereby due diligence is carried out proportionate to the nature of the materials themselves, and the circumstances under which they have been/are to be collected and provided for use. The purpose of this is to address and mitigate any potential legal and/or ethical implications of receipt and use of the materials as part of the research project, and to ensure that in doing so we align with best practice wherever possible. The overarching areas of consideration are:

• Ethical review of provenance and sourcing of the material>

• Legality of collection, transfer and use (national and international) 

Each transfer of samples is further undertaken according to a Research Collaboration Agreement or Material Transfer Agreement entered into by the Darwin Tree of Life Partner, Genome Research Limited (operating as the Wellcome Sanger Institute), and in some circumstances other Darwin Tree of Life collaborators.

## Data Availability

European Nucleotide Archive: Euplagia quadripunctaria. Accession number PRJEB64740; https://identifiers.org/ena.embl/PRJEB64740. The genome sequence is released openly for reuse. The *Euplagia quadripunctaria* genome sequencing initiative is part of the Darwin Tree of Life (DToL) project. All raw sequence data and the assembly have been deposited in INSDC databases. The genome will be annotated using available RNA-Seq data and presented through the Ensembl pipeline at the European Bioinformatics Institute. Raw data and assembly accession identifiers are reported in Table 1 and Table 2.

## Author information

Members of the University of Oxford and Wytham Woods Genome Acquisition Lab are listed here: https://doi.org/10.5281/zenodo.12157525.

Members of the Natural History Museum Genome Acquisition Lab are listed here: https://doi.org/10.5281/zenodo.12159242.

Members of the Darwin Tree of Life Barcoding collective are listed here: https://doi.org/10.5281/zenodo.12158331.

Members of the Wellcome Sanger Institute Tree of Life Management, Samples and Laboratory team are listed here: https://doi.org/10.5281/zenodo.12162482.

Members of Wellcome Sanger Institute Scientific Operations: Sequencing Operations are listed here: https://doi.org/10.5281/zenodo.12165051.

Members of the Wellcome Sanger Institute Tree of Life Core Informatics team are listed here: https://doi.org/10.5281/zenodo.12160324.

Members of the Tree of Life Core Informatics collective are listed here: https://doi.org/10.5281/zenodo.12205391.

Members of the Darwin Tree of Life Consortium are listed here: https://doi.org/10.5281/zenodo.4783558.
