# Supplementary material for: The genome sequence of the Jersey Tiger moth, Euplagia quadripunctaria (Poda, 1761)
Source: Wellcome Open Res. 2025 Feb 10;10:53. [Version 1] doi: 10.12688/wellcomeopenres.23666.1 (PMC11871433; doi:10.12688/wellcomeopenres.23666.1)
Supplement: Peer review history

# Reviewer report: Reviewer response for version 1

**DOI:** 10.21956/wellcomeopenres.26108.r118550

**Published:** 2025-02-28

**Authors:** Sivasankaran Kuppusamy

**Affiliations:** 1 Entomology Research Institute, Loyola College, Chennai, India

The authors have done the chromosome-level genome assembly of *Euplagia quadripunctaria* (Poda, 1761). They produced the comprehensive data from the genome sequence. They assigned the sex chromosome synteny also.   

**Minor comments of the Manuscript**

 Authors have sequenced the genome’s total length of 203.7 Gb using three different sequencing platforms. After the assembly they received 668.40 Mb in size. But authors haven’t observed the protein-coding genes, non-coding genes and gene transcripts.  Give reason?

 In the first line of Genome sequence report subheading the name can be given in short form like *E. quadripunctaria*

 The mitochondrial genome sequence length wasn’t mentioned in the text. It may be included in the text.

 Above all, I confirm that the manuscript meets the necessary scientific standard and is suitable for indexing

Are sufficient details of methods and materials provided to allow replication by others?

Yes

Is the rationale for creating the dataset(s) clearly described?

Yes

Are the datasets clearly presented in a useable and accessible format?

Yes

Are the protocols appropriate and is the work technically sound?

Yes

Reviewer Expertise:

Mitogenome analysis of Noctuoidea moths

I confirm that I have read this submission and believe that I have an appropriate level of expertise to confirm that it is of an acceptable scientific standard.

# Reviewer report: Reviewer response for version 1

**DOI:** 10.21956/wellcomeopenres.26108.r118551

**Published:** 2025-02-21

**Authors:** Arun Arumugaperumal

**Affiliations:** 1 Department of Biotechnology, Rajalakshmi Engineering College, Chennai, Tamilnadu, India

This is the first report of the whole genome sequence of the Jersey Tiger moth *Euplagia quadripunctaria*. The genome size was 668.4 Mbp. The assembly reported here has 31 chromosomes. The mitochondrial genome of size 15.48 kb was also reported. The authors have used long read sequencing technology. The BUSCO analysis shows 96% completeness of genome sequence. The data note can be accepted for indexing.

Are sufficient details of methods and materials provided to allow replication by others?

Yes

Is the rationale for creating the dataset(s) clearly described?

Yes

Are the datasets clearly presented in a useable and accessible format?

Yes

Are the protocols appropriate and is the work technically sound?

Yes

Reviewer Expertise:

Bioinformatics, Genomics

I confirm that I have read this submission and believe that I have an appropriate level of expertise to confirm that it is of an acceptable scientific standard.
